# Towards Long-Range RNA Structure Prediction in Eukaryotic Genes

**DOI:** 10.3390/genes9060302

**Published:** 2018-06-15

**Authors:** Dmitri D. Pervouchine

**Affiliations:** 1 Skolkovo Institute for Science and Technology, Ulitsa Nobelya 3, Moscow 121205, Russia; d.pervouchine@skoltech.ru; Tel.: +7-495-280-1481 (ext. 3925); 2 The Faculty of Bioengineering and Bioinformatics, Moscow State University 1-73, Moscow 119899, Russia; 3 Faculty of Computer Science, Higher School of Economics, Kochnovskiy Proyezd 3, Moscow 125319, Russia

**Keywords:** long-range, RNA structure, folding, RNA–RNA interaction, mutually exclusive splicing, RNA processing, polyadenylation, *Dscam*, *Nmnat*, DST

## Abstract

The ability to form an intramolecular structure plays a fundamental role in eukaryotic RNA biogenesis. Proximate regions in the primary transcripts fold into a local secondary structure, which is then hierarchically assembled into a tertiary structure that is stabilized by RNA-binding proteins and long-range intramolecular base pairings. While the local RNA structure can be predicted reasonably well for short sequences, long-range structure at the scale of eukaryotic genes remains problematic from the computational standpoint. The aim of this review is to list functional examples of long-range RNA structures, to summarize current comparative methods of structure prediction, and to highlight their advances and limitations in the context of long-range RNA structures. Most comparative methods implement the “first-align-then-fold” principle, i.e., they operate on multiple sequence alignments, while functional RNA structures often reside in non-conserved parts of the primary transcripts. The opposite “first-fold-then-align” approach is currently explored to a much lesser extent. Developing novel methods in both directions will improve the performance of comparative RNA structure analysis and help discover novel long-range structures, their higher-order organization, and RNA–RNA interactions across the transcriptome.

## 1. Introduction

Eukaryotic RNA processing is remarkably complex. Nascent pre-mRNA transcripts are spliced, edited, capped, cleaved, and polyadenylated [1]. All these events occur co-transcriptionally and are tightly coupled: Splicing affects cleavage and polyadenylation and viceversa [2,3], RNA editing can disrupt or create binding sites for splicing factors [4,5], etc., but more importantly, as RNA is being synthesized, it becomes coated by an army of RNA-binding proteins and folds into complex intramolecular structures.

The structure of RNA molecules is believed to comprise two levels: the secondary structure, which is formed by proximate regions in the primary sequence, and the tertiary structure, which also includes long-range interactions [6]. That is, the secondary structure is local, i.e., it forms between nearby sequences during Pol II elongation; in contrast, the tertiary structure is global, i.e., it builds from pre-formed helical domains of the local structure. Controversially, both terms refer to the secondary level of structure organization, in the sense that they both constitute residue interactions that are stabilized by stacking energies. The main difference between local and global structure is therefore in the number of nucleotides separating the interacting parts. Notably, long-range interactions between assembled helical domains tend to produce more pseudoknots than do local secondary structures [7]. Throughout this review, I use the term “long-range RNA structure” in the sense that refers to complementary intramolecular interactions of distant RNA regions rather than to the timing of structure formation, its topology, or 3D organization.

In vivo, the structure of a pre-mRNA is critically important for its processing. Native RNAs are folded co-transcriptionally with the aid of RNA binding proteins (chaperones) or by forming structural intermediates that help to avoid traps in dysfunctional conformations [6,8]. To date, these dynamic interactions are poorly characterized, and assays of RNA–protein interactions and transcription kinetics are just starting to emerge [9,10]. RNA-binding proteins and elongation kinetics introduce large uncertainty to the parameters of the models that are commonly used for RNA folding and represent the major source of discrepancies between computational models of long RNAs [11,12]. As a result, our ability to predict eukaryotic pre-mRNA structure is biased towards local structures, while the prediction of long-range RNA structure remains problematic from the computational point of view.

This review consists of two parts. The first part discusses the existing examples of functional long-range structures in eukaryotic RNAs and outlines molecular mechanisms related to their function. The second part targets bioinformatics readership. It summarizes the current state of the art in the field of comparative RNA structure prediction, with its advances and limitations, and discusses the perspectives and directions where it could next develop. The second part is not designed to be a complete review of all RNA structure predictions methods; thus I cite only the selected computational works that contain the most references to other papers in the field.

## 2. Instances of Long-Range RNA Structure

Functional long-range base pairings in RNAs are known throughout the tree of life [13]. They are particularly well-studied in viruses [14], including tobacco mosaic virus [15], hepatitis B and C viruses [16,17], Dengue virus [18], and human immunodeficiency virus [19]. Over the past several years, there has been an increasing number of reports on functional long-range structures in eukaryotic RNAs [20]. Table 1 provides a short list of these structures. Their functionality is mainly associated with pre-mRNA processing, usually with splicing, and more rarely with translation [21,22]. Several molecular mechanisms have been proposed for the function of these structures, with different degrees of experimental evidence [20].

The classic RNA structure probing method based on the difference in reactivities of single-stranded and double-stranded residues, even in its modern high-throughput incarnation [23], is not quite useful at long distances because it can detect whether a nucleotide is paired, but it cannot tell to which other nucleotide. While local interaction partners can be guessed from the nearby sequence, too many options arise for complementarity at long ranges. A method of photochemical cross-linking with psoralens was developed in 1979 to localize structural interactions in eukaryotic RNAs [24]. Although this method was recently implemented in high-throughput [25,26,27,28], it is not yet in common use. The most convincing assays for RNA functionality are based on double mutants, i.e., a mutation that disrupts the RNA helix and leads to the loss of function, followed by a compensatory mutation that restores base pairing and regains the function. This method is significantly more laborious because it requires introducing point mutations in constructs or to the genome, and is limited to the cases when the single-mutant state is not lethal.

Among the eukaryotic genes with functional long-range base pairings, the most known are genes with mutually exclusive exons (MXEs), of which the most fascinating example is Down’s syndrome cell adhesion molecule *Dscam* in *Drosophila* (see [29] for review). The history of *Dscam* started in 2005 when it was found that its exon 6 cluster, which consists of 48 variable exons, contains competing long-range RNA base pairings that form in a mutually exclusive way [30]. It was proposed that competing RNA structure exposes a group of exons in a loop and thereby ensures that one and only one exon is included in the mature transcript. Later, a similar splicing pattern was found also in exon 4, exon 9, and exon 17 clusters of *Dscam* [31,32,33]. However, the details of the molecular mechanism remained incomplete until many more structures were discovered in this gene, including locus control region [34] and another set of long-range structures [35]. The same principle for mutually exclusive splicing as in *Dscam* was observed in other genes, including competing long-range RNA structures in *14-3-3ζ* gene [31], bidirectional pairing control of alternative exon 4 inclusion *srp* pre-mRNAs [36], and multiple competing base pairings in *MRP1* gene [37] in *Drosophila* (see [29] for more details).

While mutually exclusive exon choice is a peculiar splicing pattern, long-range RNA structure is important for coordination of other types of alternative splicing events. To name a few, the *Nmnat* gene controls the inclusion of its alternative exon coupled with alternative polyadenylation by a pair of complementary intronic sequences in *Drosophila* [38]. Human splicing factor 1 (*SF1*) contains a long-range RNA structure in a constitutive intron preventing intron retention that leads to a lethal frameshift [20]. A cluster of six exons in human *DST* gene undergoes mutually inclusive splicing, a scenario opposite to that of mutually exclusive exons, in which either all exons in the array, or none of them are included in the mRNA. This pattern is likely due to a pair of complementary sequences, which flank the exon cluster and lead to its exclusion by forming an RNA helix that exposes the entire cluster in a loop [39].

Several mechanisms were proposed to explain the impact of long-range RNA structure on splicing [29,40]. Among them, the two major scenarios are the hindrance of a stretch of the pre-mRNA in a loop and spatial approximation of distant regulatory elements (in fact, the former causes the latter). Two mammalian genes, a kinesin superfamily member *KIF21A* and an actin regulator *ENAH*, each contain a distal intronic site that is bound by Rbfox1 and Rbfox2 in the mouse brain and Rbfox2 in human 293T cells. However, these sites act as splicing enhancers only when brought in proximity of the target exon via the formation of a long-range RNA bridge, a duplex which spans over 10Kb (hence the name) [41]. Splicing of the catalytic subunit of the human telomerase gene *TERT* also depends on the long-range RNA pairing between repeat clusters, which approximates exons 6 and 9 and suppresses exons 7 and 8, thereby promoting the so-called “minus beta” splicing [42].

Long-range RNA structures contribute to human disease, including neurological disorders and other pathologies [47]. In particular, a long-distance RNA structure that consists of three adjacent intronic RNA stems is a critical regulator of splicing in Survival Motor Neuron 2 (*SMN2*) exon 7, the skipping of which is linked to spinal muscular atrophy, a hereditary infant disease leading to early death [43,44]. Alternative splicing of human *PLP1*, a gene responsible for X-linked leukodystrophy Pelizaeus–Merzbacher disease, is also regulated by a long-distance interaction between two highly conserved complementary intronic elements [45]. Antisense oligonucleotides represent a prominent strategy for targeting such structured RNAs therapeutically, and some of them are already approved for clinical use [47,48]. In this regard, the identification of long-range RNA structures implicated in human disease becomes exceptionally important.

Several recent computational and experimental studies independently concluded that long-range base pairings in eukaryotic RNAs are abundant [20,25,26,27,28,39]. On the other hand, the examples from the short catalogue listed here (Table 1) demonstrate that long-range intramolecular base pairings are crucial for pre-mRNA processing. They must therefore represent only the tip of the iceberg, and efficient computational methods are needed to discover many more structures that are still hidden in eukaryotic genomes.

## 3. Predicting Long-Range RNA Structure

The universe of RNA structure prediction tools can be broadly divided into methods predicting intramolecular vs. intermolecular structure, on one hand, and methods based on single-sequence (de novo) vs. comparative sequence analysis on the other hand. The majority of de novo intramolecular methods implement dynamic programming for free energy minimization. Dynamic programming is effective only for unknotted structures, and its use for long RNA folding is limited because long-range interactions become shunted by local nested base pairings [39].

Although long-range RNA structure is intramolecular, it could be considered intermolecular from the prediction standpoint. Though intermolecular methods are generally as complex, many of them model RNA–RNA interactions as disassembly of the local structure followed by intermolecular hybridization, i.e., as interaction of pre-formed helical domains (see [49,50] for review and benchmark). Another possibility is to account for the contribution of pseudoknots by considering individual helices instead of base pairs, but this approach must be combined with phylogenetics [51]. At the scale of eukaryotic genomes RNA–RNA interaction prediction becomes challenging both in terms of performance and specificity because the amount of random complementarity grows with the length.

When single-sequence analysis fails, comparative methods provide a powerful alternative. The advantage of comparative methods is twofold. First, they confine the search space to evolutionarily conserved regions, which at least partly reduces the complexity and improves specificity. Second, at least hypothetically, they gain statistical power through observing compensatory changes in covarying positions [52,53,54,55,56]. These ideas, which stem from covariance models [57], have been remarkably successful in the discovery of riboswitches [58]. Among examples presented in this review, many functional long-range RNA structures were, in fact, first discovered in multiple sequence alignments and later confirmed experimentally.

In eukaryotic genomes, however, the comparative formulation becomes intricate when it meets large distances and complex organization of the genes. The conservation rates in exons and introns are fundamentally different, introns may not always be aligned, or the alignment may not be unique. While some methods use phylogenetic substitution models to fold protein-coding sequences [55,56], much less is known about comparative folding of introns and untranslated regions. Here, a reasonable solution is to combine multiple sequence alignment with RNA folding, which is the famous “simultaneous folding and aligning problem” that was first formulated in 1984 by Sankoff [59]. The Sankoff algorithm is computationally expensive, and its rigorous implementation for two sequences has the time and memory complexity $O(n^{6})$ and $O(n^{4})$, respectively, where *n* is the length of each sequence [60]. One can use it to realign an existing multiple alignment, but the depth of this realignment is limited [61]. In application to long-range intramolecular RNA structure, Sankoff method is far beyond computational capacity for n≃10,000 (see Table 1). It could be adapted for simultaneous alignment and intermolecular structure prediction for two pairs of RNA sequences with time and memory complexity of $O(n^{4})$, which is still impractical for most human introns.

Eukaryotic long-range RNA structures listed in Table 1 have a number of characteristic properties. First, they evolve under negative selection, although the rate of conservation of a structural element depends on the time in evolution when it was first acquired. After the last common ancestor, the evolution of *Dscam* went differently in *Drosophila* and in *Chelicerata* [35], while the regulatory sequence in *Nmnat* remained remarkably conserved [38]. Most of the listed structures contain uninterrupted helices of at least 12 nucleotides, many of which are surrounded by more diffuse base pairings. This is likely due to the free energy constraints to maintain long-range interaction, although the intervening sequences could also be structured since there is no apparent correlation between the stem length and the loop size. Finally, almost all examples in the table are located in syntenic regions, e.g., in introns separating orthologous exons, possibly reflecting locations related to their function and evolution.

In sum, functional long-range RNA structures have the following characteristic properties:

1. most long-range RNA structures are well-conserved;
2. the core of a long-range RNA structure is a long, nearly-perfect complementary match;
3. elements of long-range RNA structures are located in syntenic regions.

**Figure 1:**
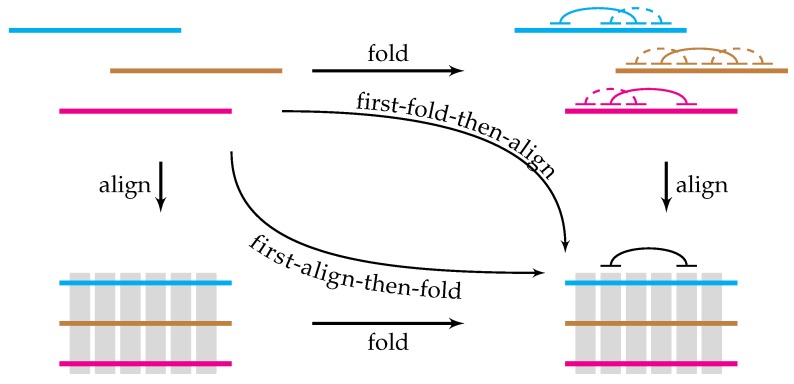
A “commutative diagram” of the alignment and folding tradeoff. Top left: unaligned RNA sequences. Bottom left: their structure-agnostic alignment; conserved regions are shown in gray. Top right: sparse folding identifies candidate helices shown as arcs. Bottom right: conserved helices are matched by structure-aware alignment or identified in a multiple sequence alignment.

The first property justifies the so-called “first-align-then-fold” limit of Sankoff’s method (Figure 1), in which a set of orthologous sequences is first aligned and the alignment is then folded. It is the most frequent approach in comparative methods [49]. By construction, it disregards the hypothetical cases of sequences that have diverged beyond recognition, but their structure has remained unchanged. Its sensitivity is limited by the quality of the input alignment, particularly by the uncertainty of aligning mutually exclusive exons that arise from genomic duplications, or by misalignment of conserved structural elements that are too short relative to the size of non-conserved background [34,62]. Apart from these special circumstances, “first-align-then-fold” is a simple, fast, and powerful approach that is used in many current comparative methods, including comparative RNA–RNA interaction prediction [52,63] and probabilistic sampling [64].

The advantage of folding a multiple sequence alignment compared to folding its consensus sequence is the covariation statistics. In general, it is possible to gain statistical power from observing covarying positions only when sequences mutate, e.g., in rapidly evolving viral genomes [54]. However, the examples from mammalian and insect genes (Table 1) show little or no variation, suggesting that functional structures evolve under strong negative selection [38]. In addition, compensatory patterns can arise not only to maintain base-pairing interactions, but also as a result of synchronized mutations that preserve binding of a common interaction partner in antisense genomic orientation [53]. An example of this is *RP11-439A17.4* long non-coding RNA, which is located in antisense to *HIST2H2BA* gene and overlaps a transcription factor binding site, which also occurs in almost all human histone genes in sense orientation, resulting in a seeming compensatory pattern [39]. Thus, the comparative approach is less efficient in the case of extreme conservation.

The second and the third property suggest the opposite, “first-fold-then-align” route (Figure 1) which is explored to a much lesser extent. Indeed, the number of folds for a single sequence taken to the power of their combinations in the multiple alignment does not appear feasible at first glance. A pioneer work on local secondary structure folded and aligned enterovirus mRNAs using phylogenetic comparison of potential stems followed by the consistency analysis of structure graph [65]. These ideas were extended to long-range structures [39], where a dramatic reduction of the fold space was achieved by considering sparse structures, i.e., ones that consist of long, nearly complementary matches. This, and the assumption that the interacting parts are located in syntenic regions, decrease the number of ways in which the helices can be aligned to the point where whole-transcriptome analyses become possible [39]. However, while this approach is quite sensitive, it does so at the expense of a high false positive rate and cannot deal with repeats and other low complexity regions.

The diagram in Figure 1 becomes commutative, i.e., predictions by the two methods coincide, when all three properties are met. Here, one unexplored and a potentially productive approach would be to match, within a certain distance limit, all standalone conserved regions that do not have protein-coding constraints and score them by how abruptly the conservation outside of them ends. It is a frequently observed pattern that non-complementary background in long-range RNA structures is washed out immediately outside of the complementary region. Another potentially useful direction is to combine comparative approaches with experimental methods that give global mapping of RNA duplexes to narrow down the space of potential complementary interactions [25,26,27,28].

## 4. Concluding Remarks

Intramolecular structure of eukaryotic RNAs is not limited to hairpins and can span thousands of bases. Recent high throughput experimental assays confirmed that distant interactions in the human transcriptome are very abundant. However, the computational identification of long-range RNA structure remains problematic because the interacting parts are separated by long distances.

The principles of computational identification of RNA structure by comparative methods span between two extremes. On one side are the so-called “first-align-then-fold” methods, which essentially look for complementary regions in multiple sequence alignments. They are powerful for well-conserved sequences, but hardly applicable to non-conserved regions in eukaryotic genes that often harbor functional RNA structure elements (Table 1). On the other side are more complex “first-fold-then-align” methods, which are applicable to non-conserved regions, but have a high false positive rate.

Characteristic features of long-range RNA structures that are outlined in this review demonstrate that, with additional assumptions, both types of methods are computationally tractable at the scale of eukaryotic genes. Further development in these both directions will expand the capabilities of comparative RNA structure analysis and lead to a discovery of many novel long-range RNA structures, their higher-order organization, and RNA–RNA interactions in the human transcriptome.

## Figures and Tables

**Table 1:** Functional long-range RNA structures in *Drosophila* and Human.

Species	Gene	Function	Length *	Spread *	References
*Drosophila*	*Dscam*	Exon 4 cluster	13	4500	[31]
	*Dscam*	Exon 6 cluster	16	11,000	[30,32,34]
	*Dscam*	Exon 9 cluster	16	14,000	[31]
	*Dscam*	Exon 17 cluster	15	1000	[33]
	*Mhc*	Exon 7 cluster	14	2500	[31]
	*Mhc*	Exon 9 cluster	14	1600	[31]
	*Mhc*	Exon 11 cluster	15	2600	[31]
	*Nmnat*	Exon 5 and polyA site	14	400	[38]
	*Atrophin*	Exon 10	16	350	[38]
	*srp*	Exon 4 cluster	21	450	[36]
	*14-3-3ζ*	Exon 5 cluster	22	1200	[31]
Human	*SF1*	Exon 10	17	100	[20]
	*ENAH*	Exon 11a	18	1800	[41]
	*DST*	Exons 47-52	15	10,000	[39]
	*SMN2*	Exon 7	8 + 7 + 8	280	[43,44]
	*PLP1*	Exon 3	10 + 5	600	[45]
	*TERT*	Exons 7 and 8	Repeat	6500	[42]
	*NEAT1*	Paraspeckle formation	N/A	10,000	[46]

(*) Length: approximate number of base pairs in complementary regions; Spread: loop size, i.e., sequence distance between complementary parts; N/A: not applicable.
